# Good recovery without decompression fasciotomy for crush syndrome caused by using a Japanese-style toilet

**DOI:** 10.1016/j.tcr.2021.100411

**Published:** 2021-02-10

**Authors:** Akinori Osuka, Daiki Miyao, Yuji Kuge, Shinji Nakajima, Yuichi Kuroki, Masashi Ueyama

**Affiliations:** Department of Trauma, Critical Care Medicine and Burn Center, Japan Community Healthcare Organization Chukyo Hospital, Nagoya, Japan

**Keywords:** Crush syndrome, Squatting position, Japanese-style toilet, Fasciotomy

## Abstract

We report a case of crush syndrome that developed while the patient was squatting to use a Japanese-style toilet. The patient was a 61-year-old male with an obese body. He was sitting on the toilet and couldn't stand up, and after a few hours, the landlord found him and called the emergency services. On presentation, the patient was hyperkalemic and in shock, and his serum creatine kinase levels rose to a maximum of 287,600 U/L. He was diagnosed with postural crush syndrome in both lower extremities due to squatting position in a Japanese-style toilet. Subjective symptoms, physical examination, and blood tests were monitored and the patient was observed. As a result, the patient could be treated conservatively without fasciotomy. Dialysis was not necessary because the fluid infusion maintained adequate urine output and corrected the hyperkalemia. Magnetic resonance imaging of both lower extremities showed multiple high-signal areas in the muscles of the bilateral thighs and lower legs. This case suggested that if the wound is closed, the peripheral pulse is palpable, and the patient's symptoms have improved, a fasciotomy should not be performed. People who are too heavy to squat may need to be careful when using this kind of toilet.

## Introduction

Crush syndrome causes renal dysfunction, rhabdomyolysis, electrolyte abnormalities and arrhythmia, and the prognosis is poor [1]. There is not much evidence on its management, especially regarding the pros and cons of fasciotomy for decompression, which remains controversial [2]. In the present study, we report a favorable outcome without performing a fasciotomy for crush syndrome of the lower extremities caused by squatting while using a Japanese-style toilet.

## Case presentation

**Fig. 1:**
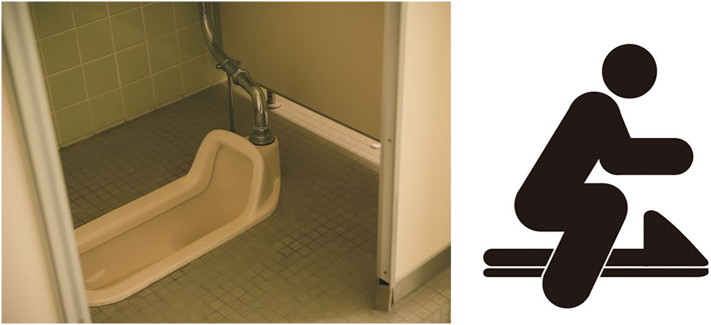
Japanese-style toilet. The left panel shows a Japanese style toilet, and the right panel is a pictogram showing how to use it. A Japanese toilet requires squatting down to defecate.

**Fig. 2:**
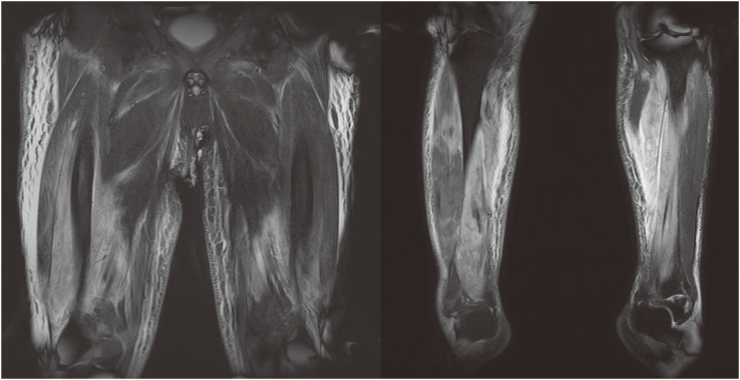
Magnetic resonance imaging of both lower extremities shows multiple high-signal areas in the muscles of the bilateral thighs and lower legs.

The patient was a 61-year-old man with a height of 166 cm and weight of 98 kg (body mass index: 36 kg/m^2^). He had a history of depression and hypothyroidism. After he was unable to stand up from a squatting posture after defecating while using a Japanese-style toilet (Fig. 1), he was found a few hours later by his landlord, who called for an ambulance. In the emergency room, his blood pressure was 81/48 mm Hg, his heart rate was 110 beats/min, and he was conscious with a clear mental state. His body temperature was 36.2 °C, respiratory rate was 35 breaths/min, and his SpO_2_ was 91% on room air. His electrocardiogram showed tent-shaped T-waves. His chief complaint was weakness of both lower extremities. Emergency ultrasound showed no deep venous thrombosis. His serum potassium was high at 7.9 mmol/L and creatine kinase (CK) was 41,116 U/L. His serum myoglobin was 267,200 ng/mL and urinary myoglobin was 600,000 ng/mL. After a rapid infusion of 1 L of saline, glucose insulin therapy, and calcium gluconate administration, his serum potassium level dropped to 6.2 mmol/dL and he had an adequate urine output, so he was admitted to the intensive care unit while waiting for dialysis with a diagnosis of crush syndrome and rhabdomyolysis. After admission, he had pain in both lower extremities, but it could be self-controlled. Palpation of both dorsal pedal arteries indicates good arterial flow. Intravenous infusion of lactated Ringer's solution was started at a rate of 300 mL/h, and total infusion volume on the first day was 5896 mL over 14 h with a urine output of 1010 mL over the same period. No vasopressors were needed to maintain his blood pressure. The next day, his CK was elevated to 287,600 U/L, but his pain was improving. His serum potassium level had fallen into the normal range, and palpation of both dorsal pedal arteries continued to indicate good blood flow. Therefore, the patient continued to receive fluid replacement and follow-up care without dialysis. Changes in his serum levels of lactate, potassium, CK, blood urea nitrogen, and creatinine over 21 days are shown in Table 1. Continuous heparin was administered to prevent deep vein thrombosis, which did not occur during the course of his hospitalization. A magnetic resonance imaging taken on day 13 showed multiple areas of inflammation in the bilateral thighs and lower legs (Fig. 2). The results for various myositis-related antibodies were negative. He was able to walk with assistance on day 25 after the injury and was transferred to a rehabilitation hospital on day 28 after the injury.

**Table 1:** Laboratory findings over 21 days.

	Days after injury						
	0	1	2	3	7	14	21
Lactate (mmol/L)	6.2	2.1	1.5	1.3	1.5	1.0	–
K (mmol/L)	7.9	4.9	5.0	4.1	3.6	3.3	3.9
CK (U/L)	41,116	287,600	152,790	81,637	8280	1005	336
BUN (mg/dL)	22	22	17	17	20	16	17
Creatinine (mg/dL)	1.91	1.77	1.48	1.48	1.29	1.09	0.93

## Discussion

This is the first reported case, to our knowledge, of crush syndrome occurring in a squatting position while using a Japanese toilet. This patient was treated without fasciotomy or dialysis, and the outcome was good. In Japan, newer buildings are switching to sit-down toilets, but many of the toilets in older buildings are still of the Japanese squatting type. People who are too heavy to squat may need to be careful when using this kind of toilet.

Crush syndrome is caused by a crush injury caused by skeletal muscle being pressed underneath a heavy object. In crush syndrome, the muscle is severely damaged and becomes swollen due to ischemic reperfusion [3], making compartment syndrome almost inevitable. Fasciotomy is the only effective treatment for acute compartment syndrome following fracture [4]. However, following crush injury, the efficacy of fasciotomy is still controversial. In post-fracture compartment syndrome, the muscle at risk can be saved by a fasciotomy, whereas in a crush injury, if treatment is delayed, the muscle has already necrotized. The complication rate of fasciotomy for crush injury is high, with the most serious complication being uncontrolled bleeding and sepsis due to wound infection [5]. There is no evidence that fasciotomy improves outcomes; rather, delayed fasciotomy was reported to result in worse physical outcomes [2]. Reports from Iran and Turkey similarly show that fasciotomy is associated with sepsis and sepsis-related death [6]. However, in the case of open wounds, cleaning and debridement are essential [7]. There seems to be no dispute that surgical decompression is necessary if the peripheral pulses of the extremities are not palpable [8]. Amputation may be required if vital signs and symptoms progressively worsen despite aggressive resuscitation [9]. However, our case suggested that if the wound is closed, the peripheral pulse is palpable, and the patient's symptoms have improved, a fasciotomy should not be performed.

## Funding

This work was supported by Grants-in-Aid for Scientific Research (grant no. 19K09413) from the 10.13039/501100001700Ministry of Education, Culture, Sports, Science and Technology of Japan.

## Declaration of competing interest

The authors have declared that no competing interests exist.
